# Clinical and radiographic outcomes of upper thoracic versus lower thoracic upper instrumented vertebrae for adult scoliosis: a meta-analysis

**DOI:** 10.1590/1414-431X20176651

**Published:** 2018-02-26

**Authors:** X. Kang, L. Dong, T. Yang, Z. Wang, G. Huang, X. Chen

**Affiliations:** Department of Bone Diseases, Hong-Hui Hospital, Xi'an Jiaotong University College of Medicine, Xi'an, China

**Keywords:** Upper instrumented vertebrae, Upper thoracic segments, Lower thoracic segments, Meta-analysis, Adult scoliosis

## Abstract

The aim of this study was to evaluate the clinical and radiographic outcomes of upper thoracic (UT) versus lower thoracic (LT) upper instrumented vertebrae (UIV) for adult scoliosis by meta-analysis. We conducted a literature search in three databases to retrieve related studies up to March 15, 2017. The preliminary screened studies were assessed by two reviewers according to the selection criteria. All analyses were carried out using the statistical software package R version 2.31. Odds ratios (OR) with 95% confidence intervals (CI) were used to describe the results. The I^2^ statistic and Q statistic test were used for heterogeneity assessment. Egger's test was performed to detect publication bias. To assess the effect of each study on the overall pooled OR or standardized mean difference (SMD), sensitive analysis was conducted. Ten trials published between 2007 and 2015 were eligible and included in our study. Meta-analysis revealed that the UT group was associated with more blood loss (SMD=0.4779, 95%CI=0.3349-0.6209, Z=6.55, P*<*0.0001) and longer operating time (SMD=0.5780, 95%CI=0.1971-0.958, Z=2.97, P=0.0029) than the LT group. However, there was no significant difference in Oswestry Disability Index, Scoliosis Research Society (SRS) function subscores, radiographic outcomes including sagittal vertical axis, lumbar lordosis, and thoracic kyphosis, length of hospital stay, and revision rates between the two groups. No evidence of publication bias was found between the two groups. Fusion from the lower thoracic spine (below T10) has as advantages a shorter operation time and less blood loss than upper thoracic spine (above T10) in posterior long-segment fixation for degenerative lumbar scoliosis.

## Introduction

Adult spinal deformities (ASDs) are complex pathologies associated with a broad range of clinical and radiological presentation. The prevalence of ASD has been reported as high as 60% in the general population older than 60 years (1). Restoring and maintaining spinal balance are the key points for the successful surgical treatment of adults with spinal deformity. Conservative treatment includes physical therapy, facet blocks, nonsteroidal anti-inflammatories, nerve root or epidural injections and oral narcotic medications. However, conservative management is not always satisfactory in adult scoliotic patients (2). With the growth of the aging population, surgical management is becoming more frequent and aggressive, which might result in several complications in patients.

Recent studies have suggested that a solid arthrodesis is necessary to arrest deformity progression, and maintain correction for achievement of well-balanced spine (3,4). Long lumbar instrumented fusions have been described for ASD. Long fusion from the sacrum to the thoracic spine is a common surgical treatment option to correct sagittal imbalance. When deciding on the upper instrumented vertebra (UIV) for fusion, surgeons have to face the difficult decision in choosing a proper site between the upper thoracic spine (UT) or lower thoracic spine (LT).

The choice of an UIV site in spinal deformity is characterized by significant variability. Several indications are related to UT stopping points (T2-T5) such as thoracolumbar kyphosis, coronal and/or sagittal imbalance, large coronal curves in the thoracic spine, and osteoporosis, whereas lower thoracic (LT) points (T10-T12) are generally used for those curves that are well balanced (5,6). There are also several indications for extending fixation and fusion for the LT spine including thoracic hyperkyphosis, structural scoliosis, osteoporosis, more severe coronal and sagittal plane decompensation, and thoracolumbar junctional kyphosis (7,8). Previous studies have reported that patients treated with UIV in LT segment are associated with a higher prevalence of pseudarthrosis, severe blood loss, and longer operative time and hospital stay (5). However, there is little information to make an informed decision in the choice of UIV fusion site after long instrumented fusion for adult patients with scoliosis.

Currently, there have been some studies comparing the clinical and radiographic outcomes between upper thoracic spine (UT group) and lower thoracic spine (LT group) fusion sites. However, the results are still controversial. Thus, this meta-analysis of the literature assessed the UT and LT sites in long fusion for ASDs.

## Material and Methods

This review is reported according to the Preferred Reporting Items for Systematic Reviews and Meta-Analyses (PRISMA) guidelines (9).

### Search strategy

We conducted a literature search for relevant studies in Pubmed, Embase and Cochrane library up to March 15, 2017. The key words for searching were “adult scoliosis”, “spinal deformity”, “long fusion”, “proximal fusion”, and “upper instrumented vertebra”. Reference lists of retrieved articles and relevant reviews were hand searched. English publications were included, and no study design was restricted.

### Study selection

Two reviewers initially screened the citation titles and abstracts. The full text versions of any study of potential relevance were then screened independently in triplicate. Disagreements were resolved through discussions. If discrepancies still existed, we sought the opinions of two other researchers for further discussion.

To be considered for inclusion, studies had to meet the following criteria: 1), assess adult patients with scoliosis or spinal deformity; 2), compare UT (experimental group) with LT (control group) upper instrumented vertebrae.

Exclusion criteria were: 1) patients with scoliosis due to neuromuscular, congenital, trauma, or paralytic etiologies; 2) studies with difference intervention times; 3) conferences, letters, commentaries, editorials, and reviews. When multiple publications existed for the same study, we included the most comprehensive report or the publication with more data.

### Data extraction and quality assessment

Two authors independently extracted the data, which were recorded in a standard spreadsheet. Disagreements were resolved through discussions with other researchers. Extracted data included publication year, settings, study design, number of patients, age and duration of follow-up.

We used the Newcastle-Ottawa Scale to assess the methodological quality of included studies. The scale assessed three aspects of study methods: the selection of study groups (range 0-4), the comparability of groups (range 0-2), and the quality of outcome ascertainment (range 0-3). The total score ranged from 0 to 9, and an acceptable methodological design is reflected by a score of more than 5 (10).

### Statistical analysis

All analyses were carried out using the statistical software package R version 2.31 (R Foundation for Statistical Computing, China) with functions metacont, metabin and metabias, etc. (R package: meta) (11). Odds ratios (OR) with 95% confidence intervals (CI) were used to describe the results for dichotomous variables and standardized mean difference (SMD) with 95%CI for continuous results. We assessed heterogeneity by means of I^2^ statistic and Q statistic test, which reflects the amount of heterogeneity between studies over and above the sampling variation and is robust considering the number of studies and choice of effect measure. If the I^2^ and Q statistic indicated considerable heterogeneity (I^2^ >50% and/or P-value of Q statistic test <0.05), we combined the summary measures across the studies using random effects, otherwise, fixed model effect was used (I^2^ <50% and P-value of Q statistic test <0.05). Egger's test was used to detect publication bias. The sensitive analysis was achieved by repeating the meta-analysis omitting one individual study at a time to assess the effect of each study on the overall pooled OR or SMD (12).

## Results

### Search result

**Figure 1.**
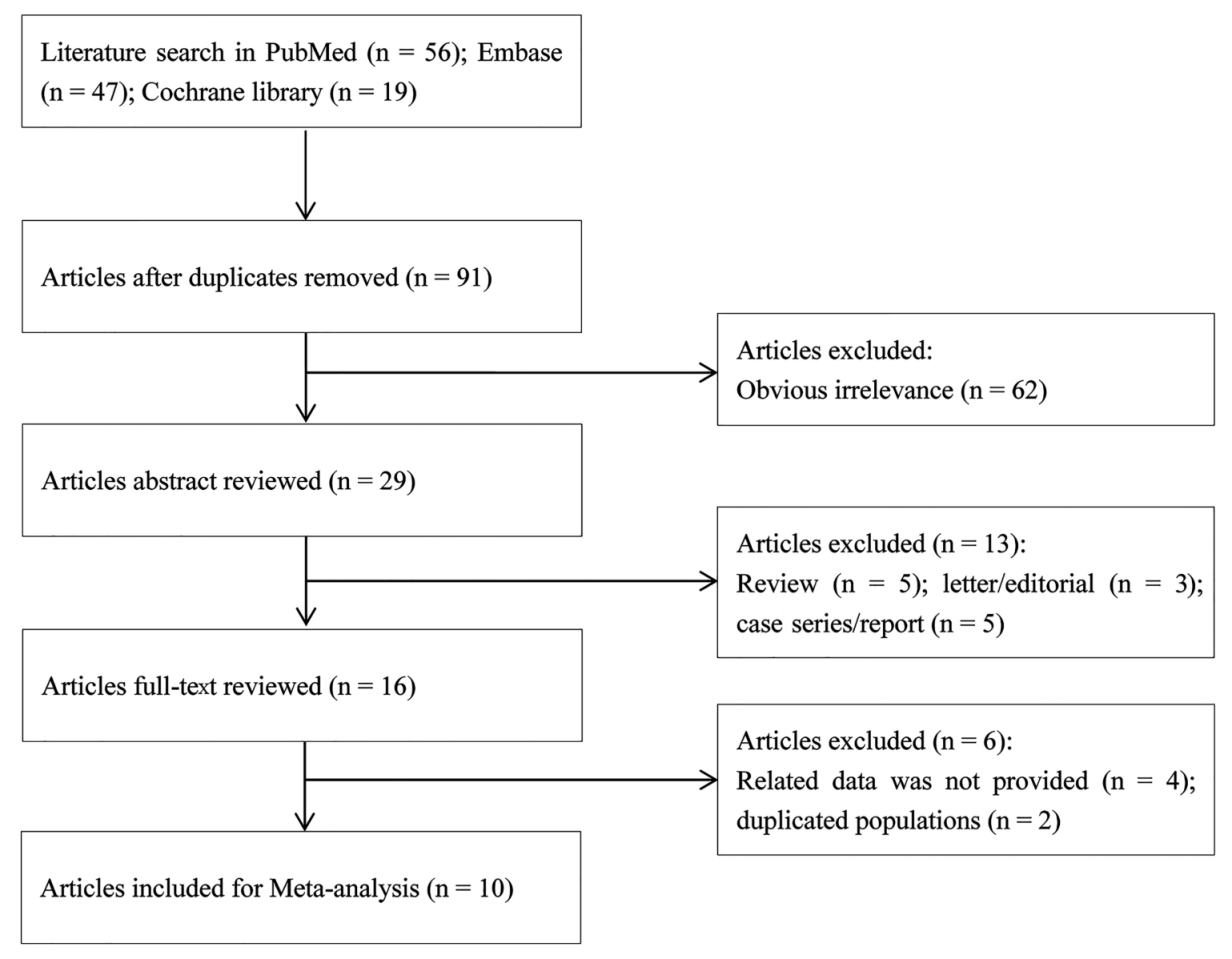
Literature search and study selection.

Figure 1 shows the flow diagram of the meta-analysis strategy. In total, 122 references were identified as potentially relevant studies, (56 references from PubMed, 47 references from Embase, and 19 references from Cochrane library). Then, we excluded 31 duplicate publications and 62 obvious irrelevance. After full-text review of the remaining studies (n=29), 19 articles were excluded because of review references (5 references), letter/editorial (3 references), case series/reports (5 references), duplicated populations (2 references), and they did not provide related data (4 references). Finally, 10 articles related to the clinical and radiographic outcomes of upper thoracic versus lower thoracic upper instrumented vertebrae for adult scoliosis were included in this meta-analysis.

The included 10 trials were from 4 countries (Korea, Japan, USA, China) and published between 2007 and 2015 (Table 1) (5–7,13–19). Mean trial duration was 24.8 weeks (ranged from 12 to 36 weeks). The quality for all 10 studies was high (>5), and the median quality score was 6, with a range of 5-8. These results showed that the observational studies were of good quality.

**Table 1.** Characteristics of included studies on posterior long-segment fixation for degenerative lumbar scoliosis

Study (year)	Ref	Country	Design	UT *vs* LT			Quantity score
				Number	Age	Fusion level	
Cho KJ (2013)	13	Korea	Retrospective	22/29	64.6/64.6	T9-T10/T11-T12	6
Fujimori T (2014)	14	Japan	Retrospective	31/49	60/62	T1-T6/T7-T12	6
Ha Y (2013)	15	USA	Retrospective	22/67	64.1/64.2	T2-T5/T9-L1	7
Kim HJ (2014)	6	USA	Retrospective	91/107	60.9/62	T1-T6/T9-L1	6
Kim YJ (2007)	7	USA	Retrospective	37/49	51.9/57.4	T9-T10/T10-T11	7
O'Shaughnessy BA (2012)	5	USA	Retrospective	20/38	55.4/55.9	T2-T5/T9-T12	8
Scheer JK (2015)	16	USA	Retrospective	81/84	60.3/59.6	T1-T6/T9-L1	5
Sciubba DM (2015)	17	USA	Retrospective	64/70	60.4/62	T1-T6/T9-L1	6
Yagi M (2013)	18	Japan	Retrospective	17/15	48.7/53.7	T1-T6/T8-T12	7
Zhu Y (2015)	19	China	Retrospective	22/33	56.2/59.4	T9-T10/T11-T12	6

UT: upper thoracic spine; LT: lower thoracic spine.

### Function score

**Figure 2.**
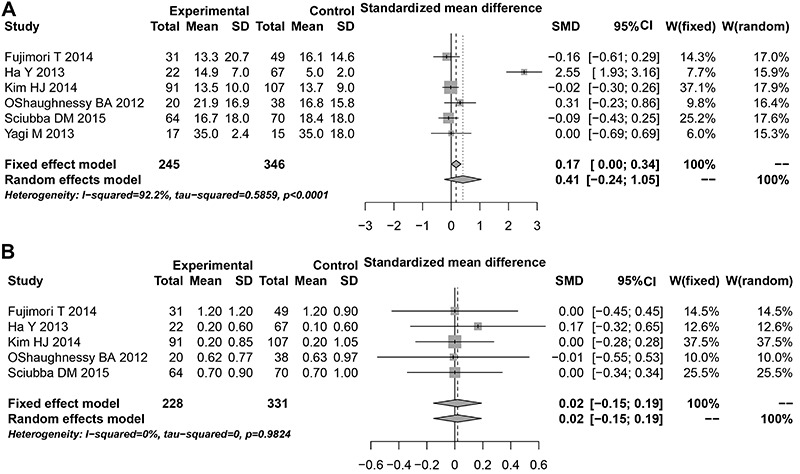
Forest plots for Oswestry disability index (*A*) and Scoliosis Research Society (SRS) function subscores (*B*). UT (experimental group): upper thoracic; LT (control group): lower thoracic. See Table 1 for reference numbers of cited studies.

Only 6 studies with 591 patients examined the change of Oswestry disability index (ODI). Meta-analysis showed that there was no significant difference in ODI score (Q=64.00, P<0.01, I^2^=92.2%, random effects model, SMD=0.4069, 95%CI=-0.2388-1.0526, Z=1.24, P=0.2168) between UT and LT groups. Egger's test (t=1.2381, P=0.2834) showed no evidence of publication bias (Figure 2A).

### SRS function subscores

Five studies reported the change of Scoliosis Research Society (SRS) function subscores in 559 patients. Meta-analysis revealed that there was no significant difference in the change of SRS function subscores (Q=0.40, P*=*0.9824, I^2^=0%, fixed effects model, SMD=0.0197, 95%CI=-0.1514-0.1908, Z=0.2258, P*=*0.8213) between UT and LT groups. Egger's test (t=0.7817, P=0.4914) showed no evidence of publication bias (Figure 2B).

### Surgical outcome

**Figure 3.**
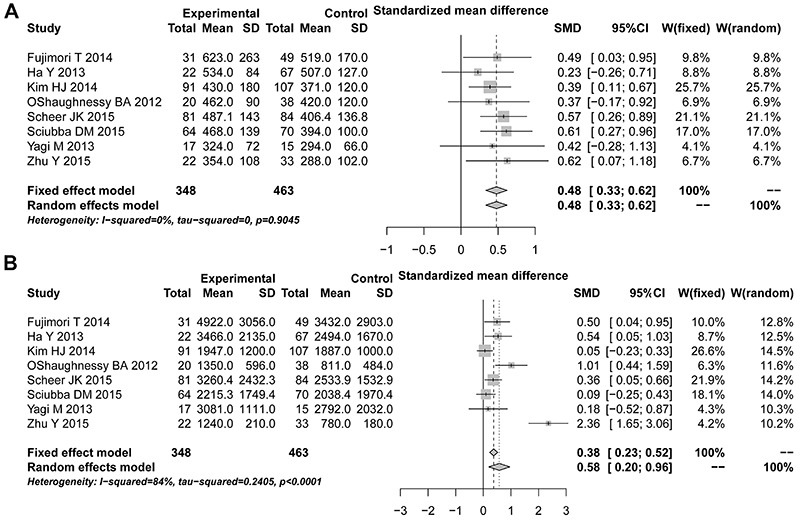
Forest plots for surgical outcome based on the of operating time (*A*) and blood loss *(B*) outcomes. UT (experimental group): upper thoracic; LT (control group): lower thoracic. See Table 1 for reference numbers of cited studies.

Eight studies with 811 patients reported the outcomes of blood loss and operating time. Meta-analysis revealed that UT group was associated with more blood loss (Q=2.78, P*=*0.90, I^2^=0%, fixed effects model, SMD=0.4779, 95%CI=0.3349-0.6209, Z=6.55, P*<*0.0001) and longer operating time (Q=43.63, P<0.01, I^2^=84.0%, random effects model, SMD=0.5780, 95%CI=0.1971-0.958, Z=2.97, P=0.0029) than LT group. Egger's test (t_blood loss_=-0.42028, *P*_blood loss_=0.6889; t_operating time_=2.5513, *P*_operating time_=0.05341) showed no evidence of publication bias (Figure 3).

**Figure 4.**
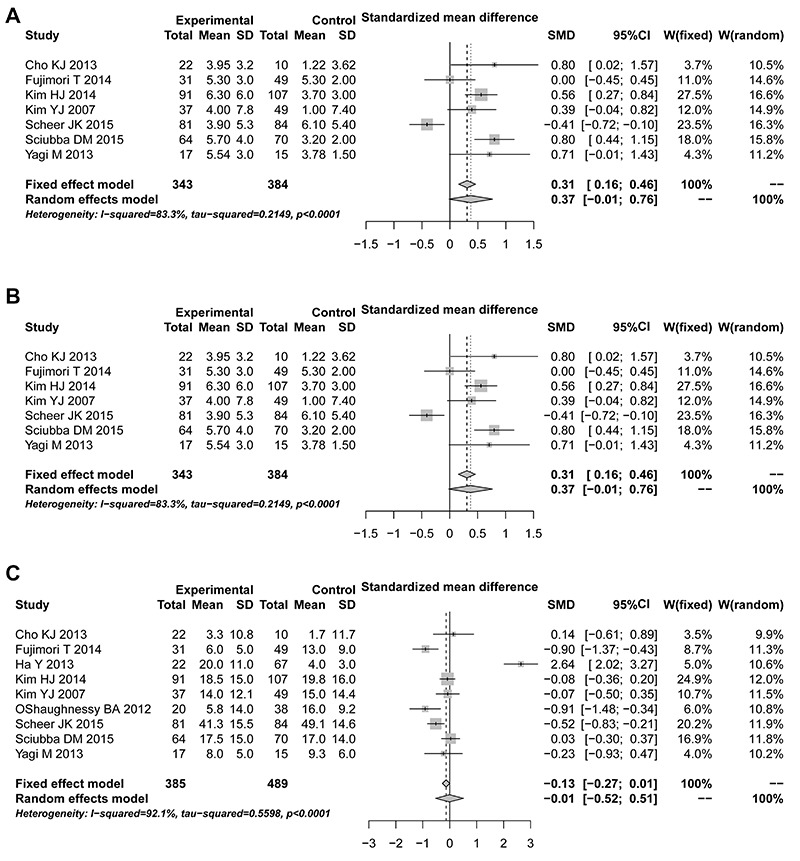
Forest plots for radiographic outcomes based on sagittal vertical axis (*A*), lumbar lordosis (*B*), and thoracic kyphosis (*C*). UT (experimental group): upper thoracic; LT (control group): lower thoracic. See Table 1 for reference numbers of cited studies.

Nine studies were available for assessing the radiographic outcomes. Meta-analysis demonstrated that there was no significant difference in sagittal vertical axis (SVA; Q=35.84, P<0.01, I^2^=83.3%, random effects model, SMD=0.3742, 95%CI=-0.0136-0.7620, Z=1.89, P*=*0.0586), lumbar lordosis (LL; Q=13.79, P=0.03, I^2^=56.5%, random effects model, SMD=-0.1320, 95%CI=-0.3943-0.1303, Z=0.99, P=0.3239), thoracic kyphosis (TK; Q=100.82, P<0.01, I^2^=92.1%, random effects model, SMD=-0.0068, 95%CI=-0.5245-0.5109, Z=0.03, P=0.9795) between UT and LT groups. Egger's test (t_SVA_= 0.58193, *P*_SVA_=0.5859; t _LL_=-1.5849, *P*_LL_=0.1738; t_TK=_0.69035, *P*_TK_=0.5122) showed no evidence of publication bias (Figure 4).

### Length of hospital stay

**Figure 5.**
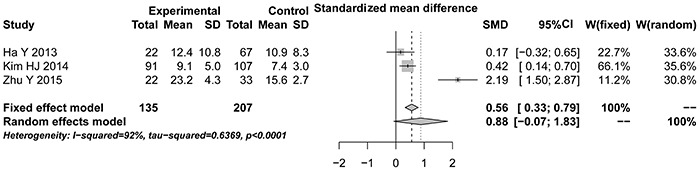
Forest plots for length of hospital stay. UT (experimental group): upper thoracic; LT (control group): lower thoracic. See Table 1 for reference numbers of cited studies. UT

Three studies provided the data for hospital stay. Meta-analysis showed that there was no significant difference in length of hospital stay (Q=100.82, P<0.01, I^2^=92.1%, Random effects model, SMD=0.8792, 95%CI=-0.0692-1.8276, Z=1.82, P*=*0.0692) between UT and LT groups. Egger's test (t=0.94534, P=0.5179) showed no evidence of publication bias (Figure 5).

### Revision rates

**Figure 6.**
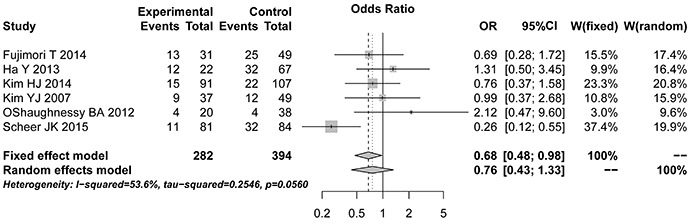
Forest plots for revision rate. UT (experimental group): upper thoracic; LT (control group): lower thoracic. See Table 1 for reference numbers of cited studies.

Six studies reported the rate of revisions on 676 patients. Meta-analysis revealed that there was no significant difference in revision rate (Q=10.78, P=0.0560, I^2^=53.6%, Random effects model, OR=0.7591, 95%CI=0.4338-1.3283, Z=0.97, P=0.3348) between UT and LT groups. Egger's test (t=2.329, P*=*0.08034) showed no evidence of publication bias (Figure 6).

### Sensitive analysis

We evaluated the effect of each study on the pooled results by excluding a single study sequentially. As a result, no change was observed concerning the analysis of ODI, SRS function subscores, operating time, blood loss, LL, and revision rate, which validated the rationality and reliability of our analysis. However, with regard to the outcome of SVA, TK and hospital stay, a single study may influence the results (See Supplmentary Figures S1-S9).

## Discussion

The long lumbar instrumented fusions are the main treatment strategy for patients with degenerative scoliosis, although there is little information about the use of UT and LT sites for UIV fusion. In this study, we performed a meta-analysis to pool the results of previous studies that reported the outcomes between UT and LT as sites of UIV in adult patients with spinal deformity. Our results showed that UT groups were associated with more blood loss and longer operating time than LT groups. However, there were no significant differences in ODI, SRS function subscores, radiographic outcomes including SVA, LL and TK, length of hospital stay, and revision rates between the two groups.

Problems seen in the proximal segments after long fusion for degenerative lumbar deformity consist of 1) proximal adjacent segment degeneration, 2) compression fracture proximal to the fusion mass, or 3) screw failure at the uppermost instrumented vertebra. Proximal adjacent segment degeneration can be detected by the following findings: 1) progressive narrowing of disc height, 2) progressive decrease in lordosis or increase in kyphosis, 3) osteophyte formation and sclerosis of adjacent endplate, or 4) translation in coronal or sagittal planes (20).

Therefore, during the UIV surgery for fusion, it is of great importance for the surgeons to determine the site of UIV. Kim et al. (21) found that the choice of UIV had the similar effect on spinal structure stability as the proximal segments degeneration. Based on our blood loss and operating time results, the choice of UIV should be at the LT segment. Clinically, the fusion for degenerative lumbar deformity should meet the following conditions (22): 1) the proximal spine should be sited in the stable region, with the coronal vertical wheel base of less than 2 cm; 2) the proximal vertebrae should be corrected in a normal vertical plane; 3) the fixed adjacent segment should not be sited in the intervertebral disc or degenerated intervertebral joint; 4) the UIV should not be rotated; and 5) the adjacent segment should be stable. Kwon et al. (23) suggested that the T1-T10 could be regarded as a stable structure because of the protection of the thorax, whereas the T11 and T12 are relatively not stable, therefore the long-segment UIV should not be located at T11 or T12 to avoid stress concentration.

However, our meta-analysis has the following limitations that must be taken into account. First, a single study may influence the whole results in the sensitivity analysis for the SVA, TK and hospital stay, which suggested our study was not powerful and stable. Second, our results were based on unadjusted estimate; outcomes that are more accurate should result from adjustments for other confounders such as gender, age, body mass index, etc. Dangelmajer et al. (24) have reported that the age and severity of the deformity are associated the clinical outcomes of minimally invasive spine surgery for patients with degenerative lumbar scoliosis. In our study, the age of patients ranged from 48.7-64.6 years, which might affect the results of our meta-analysis. Besides, we did not analyze the severity of the deformity because of unclear information from the included studies. Third, potential publication bias is very likely, in spite of no evidence being obtained from our statistical tests. Fourth, only English language reports were included and consequently we may have missed data from important studies published in other languages. Moreover, our report is limited by short follow-up and small sample size. However, we have recently completed reanalysis of most of these patients (unreported data) with minimal follow-up of 4 years and found similar results. Ultimately, longer follow-up of a larger cohort (especially randomized clinical trials) will be necessary to corroborate these preliminary results.

In conclusion, fusion from the lower thoracic spine (below T10) is as effective as fusion from the upper thoracic spine (above T10) in posterior long-segment fixation for degenerative lumbar scoliosis, with the advantages of shorter operation time and less blood loss. In addition, spinal sagittal deformity correction can be improved.

## Supplementary material

Click here to view [pdf].
